# Applying the theory of planned behavior for prediction of oocyte donation intention among Iranian women

**DOI:** 10.1186/s12884-023-05836-7

**Published:** 2023-07-24

**Authors:** Hanie Balochi, Fatemeh Hadizadeh-Talasaz, Narjes Bahri, Fatemeh Mohammadzadeh

**Affiliations:** 1 Student Research Committee, Faculty of Medicine, Gonabad University of Medical Sciences, Gonabad, Iran; 2 Department of Midwifery, School of Nursing and Midwifery, Mashhad University of Medical Sciences, Mashhad, Iran; 3 Department of Midwifery, Faculty of Medicine, Social Determinants of Health Research Center, Gonabad University of Medical Sciences, Gonabad, Iran; 4 Department of Epidemiology & Biostatistics, School of Health, Social Development and Health Promotion Research Center, Gonabad University of Medical Sciences, Gonabad, Iran

**Keywords:** Intention, Oocyte donation, Perceived behavioral control, Subjective norms

## Abstract

**Background:**

Oocyte donation is a complex and multifaceted behavior in women. Due to the acute deficiency of donated oocytes, it is necessary to identify the factors affecting the desire to donate. Planned behavior theory is a suitable model for explaining and predicting behavior in many behavioral domains. The aim of this study was to predict oocyte donation intention using the theory of planned behavior.

**Methods:**

This cross-sectional study was performed on 556 women who were covered by the comprehensive health service centers in Gonabad and Neyshabour cities in 2020. Sampling was performed by the two-step method in Gonabad city and by the convenience sampling method in Neyshabour city. Data were collected online using researcher-made questionnaires included oocyte donation awareness questionnaire and oocyte donation intention questionnaire (based on the TPB constructs). The validity of the questionnaires was confirmed by face and content validity and its reliability was confirmed by Cronbach's alpha coefficient. Data were analyzed in SPSS software (version 16) using hierarchical linear regression. The statistical significance was considered as *P* < 0.05.

**Results:**

The results showed that there was a significant positive correlation between the oocyte donation intention with all constructs of planned behavioral theory (*P* < 0.050). Planned behavior theory constructs explained 47% of the variance of oocyte donation intention. In addition, constructs of perceived power (β = 0.461), control beliefs (β = 0.154) and normative beliefs (β = 0.125) were the strongest predictors of oocyte donation intention, respectively.

**Conclusion:**

Planning and implementing educational courses according to the constructs of the theory of planned behavior can be a great step toward the elimination of oocyte deficiency and infertility problems. Also performing of intervention or training strategies in the field of oocyte donation requires the enhancement of social norms and perceived behavioral control in the population under study.

## Introduction

Infertility is a public reproductive health problem [1]. Both infertility and its treatment can be very stressful experiences [2] and affect one’s health profoundly [3]. Almost 10% of the world’s population suffers from infertility and its prevalence in Iran is 20.2% [2]. Numerous methods have been adopted to help infertile couples, including the use of donated oocytes [1], which is a popular and successful treatment among infertile women [4].

Infertile couples are increasingly using donated oocytes to overcome infertility [5], and the demand for oocytes is rising worldwide [6]. The main motivation for oocyte donation is the women’s inability to conceive using their gametes due to such factors as poor oocyte quality after several unsuccessful in vitro fertilization (IVF) attempts, low or lack of ovarian reserve due to maternal age or premature ovarian failure. Oocyte donation can also be suggested for women with an inherited genetic disease to prevent the transmission of the disorder to the next generation [7].

In Iran, oocyte donation is allowed based on the fatwa of some authorities, but in all the fatwas, it is prohibited to do forbidden preparations (unnecessary look and touch) [8].

There has been a shortage of donors in many countries [9] and the selection of a suitable donor has become a challenge for infertile couples due to the mismatch between supply and demand [10]. Sometimes the demand for donated oocytes exceeds the supply in many countries around the world [9, 10].

Shortage of oocyte donors is a major concern, and although far from ideal, in countries such as the United Kingdom, oocyte sharing models have been introduced in clinics where infertile patients donate their oocytes in exchange for subsidized fertility treatment to meet the growing demand for oocytes. Some studies have indicated that although the attitude toward oocyte donation is positive, the intention to donate is lacking [11].

Oocyte donation is a complex behavior that can be influenced by various factors. Therefore, it is of utmost importance to identify the effective factors associated with the willingness to donate to plan interventions and increase the number of donors accordingly [10].

Considering the worldwide shortage of oocyte donors, several studies have examined various factors that may influence the decision to donate oocytes in the general population of women [11]. Many factors, such as demographic characteristics, level of education, and socioeconomic status (sociodemographic) may affect women’s decision to participate in oocyte donation programs [7]. The community’s ignorance of assisted reproductive techniques is another cause of donor shortage. Moreover, the lack of donors may also be attributed to the fact that oocyte donation may be in contrast with socio-cultural and traditional norms of a given community; therefore, the negative attitudes toward donation methods lead to insufficient adoption of these methods [10]. Factors such as the possibility to receive professional consulting, talking to women who had similar donation experiences, short treatment periods, and altruistic and financial motives increase the willingness to donate [12, 13]. In some countries, such as the United Kingdom, oocyte donation is regarded as a kind of organ or tissue donation. In these countries, oocytes cannot be bought or sold and can only be donated for free [14].

Health education specialists use a variety of patterns and theories to explain health-related behaviors. In this regard, one of the most widely used theories for planning effective interventions is the theory of planned behavior (TPB). According to this theory, behavioral intention is the direct determinant of behavior. In other words, the stronger the intention to perform a behavior, the more likely the behavior will be performed [15].

Intention to perform a behavior is predicted by three factors, including attitude toward behavior, subjective norms, and perceived behavioral control. Attitude reflects a person’s positive and negative feelings toward a behavior, which is determined by two factors, including individual beliefs and behavior evaluation. Subjective norm refers to the influence of important individuals or organizations on individual behavior, which is comprised of two components of normative beliefs and motivation to comply. Perceived behavioral control refers to a degree of difficulty associated with performing a behavior and is comprised of two components of control beliefs and perceived power [16, 17].

In a study conducted in Birmingham, USA., in 2009, the TPB was reported as an effective model to predict oocyte donation behavior. Also in this study, the three components of attitude toward oocyte donation, attitude toward oocyte donation outcomes, and subjective norms could directly predict the oocyte donation intention [11]. In another study performed in Birjand, Iran (2016), the constructs of perceived behavioral control and attitude were the best predictors of intention to resolve infertility problems, respectively [18].

Considering the current shortage of oocyte donors and since intention is one of the predictors of behavior, therefore this study aimed to determine the factors that predict the oocytes donation intention among women using the theory of planned behavior as a theoretical framework.

## Methods

This cross-sectional study was performed on 556 women who were covered by the comprehensive health service centers in Gonabad and Neyshabour cities of Iran, from August to November 2020.

Among the cities of Razavi Khorasan province (located in the northeast of Iran), two cities of Gonabad and Neyshabour were randomly selected. The reason for choosing two cities was to increase the generalizability of the study. The study protocol was approved by the Ethics Committee of Gonabad University of Medical Sciences, Gonabad, Iran (IR. GMU. REC.1397.132).

Inclusion criteria included women in the age range of 20–35 years, residence in Gonabad and Neyshabour cities of Iran, willingness to participate in the study, married women or women with a history of marriage, access to social networks such as Telegram and WhatsApp, and lack of mental disorders based on the person’s self-expression (this can affect one’s responsiveness). However, those who were unwilling to continue participation were excluded from the study. The sample size was determined to be 549, using G * Power software (version 3.1.9.2) and linear regression, considering the confidence level of 0.99, test power of 0.99, the number of studied predictor variables (approximately 25 variables), and an effect size of f^2^ = 0.10. However, the sample size was increased to 597, considering an attrition rate of 10%. The post hoc analysis was performed to ensure the adequacy of the sample size. Among the samples with inclusion criteria, 556 people agreed to participate in the study and completed the questionnaires. The rate of attrition was about 7 percent.

Data collection tools included a demographic and midwifery profile questionnaire, oocyte donation awareness questionnaire, and oocyte donation intention questionnaire. The demographic and midwifery profile questionnaire included items on age, education, occupation, monthly family income, history of medical diseases, number of previous pregnancies and deliveries, history of infertility in oneself or first-degree relatives, history of using fertility donation methods, and donation history information. Oocyte donation awareness questionnaire was a researcher-made questionnaire and included 12 items. Each item in this questionnaire had three options (correct, incorrect, I do not know) which evaluated women’s awareness of oocyte donation, advantages, disadvantages, side effects, and prohibited usage. Each correct answer was scored 1 and each “incorrect” or “I do not know” answer was scored 0.

The questionnaire’s minimum and the maximum total scores were 0 and 12, respectively. A higher score indicated good awareness of oocyte donation. The total scores between 0–2.9, 3–5.9, 6–8.9, and 9–12 indicated very low, low, average, and high awareness of oocyte donation, respectively. The oocyte donation intention questionnaire was a researcher-made questionnaire that was developed based on the TPB constructs and divided into four sections, including 1) attitude towards behavior (18 items in two parts of behavioral beliefs and evaluation of behavioral consequences), 2) subjective norms (12 items in two parts of normative beliefs and motivation to comply), 3) perceived behavioral control (26 items in two parts of control beliefs and perceived power), and 4) behavioral intention (5 items). This questionnaire had a total of 61 items that were designed based on a 5-point Likert scale.

Scoring of items ranged from completely disagree (score 1) to completely agree (score 5). In some items, scoring was done in reverse. The minimum and maximum scores on this scale were 61 and 305, respectively. A higher score indicated a greater oocyte donation intention.

The validity of the oocyte donation awareness questionnaire and oocyte donation intention questionnaire was ensured by determining their formal and content validity. To evaluate the face validity, the questionnaire was completed by 10 persons with similar characteristics to those in the target group (they met the inclusion criteria), and ambiguous parts and difficult words and phrases were modified based on their opinion. The content validity of the questionnaire was evaluated qualitatively and quantitatively. To qualitatively evaluate the validity of the content, the questionnaire was distributed among 10 experts (doctorate in reproductive health or health education and a number of gynecologists) and they were asked to give their opinions about the questionnaire. The quantitatively evaluate content validity was done using coefficients of content validity ratio (CVR) and content validity index (CVI).

Phrases with CVR above 0.62 (based on the evaluation of the 10 experts) and CVI above 0.79 were retained. This led to the deletion of three items. Moreover, 20 study participants were asked to complete the oocyte donation awareness questionnaire and oocyte donation intention questionnaire to evaluate the reliability of the questionnaires, and Cronbach’s alpha coefficient was calculated subsequently. Cronbach’s alpha coefficient for the oocyte donation awareness questionnaire was determined at 0.84 which indicates reliability. Cronbach’s alpha coefficient for the whole oocyte donation intention questionnaire and each construct of the questionnaire was higher than 0.7.

Sampling was performed by the two-step method in Gonabad city and by the convenience sampling method in Neyshabour, Iran. Therefore, several centers were selected at random based on the list of comprehensive health service centers in Gonabad, Iran. In the next stage, from the prepared list, a number of women who were covered by the respective health center were selected according to the center population using a simple random method. The researcher then explained the objectives of the study through a phone conversation to the selected women who met the inclusion criteria, and they were provided with the required information and instructions about the questionnaires.

The questionnaires were created through Porsline software (an online questionnaire design software) and sent to the individuals online through social network applications, such as Telegram and WhatsApp, emphasizing the confidentiality of information. The respondents were then asked to carefully complete and submit the respective questionnaires. At the beginning of the questionnaire, participants studied the objectives of the study, and if interested to participate in the study, they could open the questionnaire by clicking the option “I agree”. Logging in to web pages and opening the questionnaire indicates participants' satisfaction with participating in the study. The respondents could quit at any time during the completion of the questionnaire. The reason for the online completion of the questionnaires was to follow health protocols to prevent the spread of the disease following the outbreak of COVID-19.

Data were analyzed using SPSS software (Version 16.0). The quantitative and qualitative variables were presented by mean ± standard deviation (SD) as well as absolute and relative frequency, respectively. The normal distribution of quantitative variables was evaluated using the Kolmogorov–Smirnov test.

Spearman correlation test was used to evaluate the correlation between TPB components and oocyte donation intention. Hierarchical linear regression was used to identify predictors of oocyte donation intention (based on TPB components) and control the effect of other variables.

Therefore, the relationship between individual variables and the oocyte donation intention score was initially investigated using simple linear regression, and variables with *P* < 0.15 were entered into the multiple linear regression model [19]. The TPB components were entered into block 1 and other variables were entered in block 2 of the model. The assumptions of the linear hierarchical models, including the normality of the residuals, the homogeneity of variance, and the independence of the residuals, were evaluated using the Kolmogorov–Smirnov test, standardized residual graphs versus predicted values, and the residual time series diagram.

Multiple alignments were also assessed using the variance inflammation factor (VIF < 5) [20]. Bilateral *p*-value less than 0.05 (*P* < 0.05) was considered statistically significant.

## Results

Out of all completed questionnaires, 556 questionnaires were complete and analyzed. The mean age of participants were 29.58 ± 4.60 years. In terms of the job, 66% of the study participants were housewives and did not smoke a cigarette and/or hookah. Furthermore, 43.5% and 45.1% had one delivery and normal delivery, respectively. The other characteristics of the participants have shown in Table 1.

**Table 1:** Demographic and obstetric characteristics of the study participants

Variable		N (%)
Educational level	Diploma or below	205 (36.8)
	Associate or Bachelor degree	305 (54.9)
	Master degree or higher	46 (8.3)
Marital status	Married	547 (98.4)
	divorced	9 (1.6)
Family income level	less than enough	98 (17.6)
	enough	433 (77.9)
	More than enough	25 (4.5)
Using contraception now	Yes	345 (62.1)
	No	211 (37.9)
History of underlying disease	Yes	28 (5)
	No	528 (95)
History of infertility	Yes	41 (7.4)
	No	515 (92.6)
History of using assisted reproductive techniques	Yes	30 (5.4)
	No	526 (94.6)
History of infertility in your first-degree relatives or spouse (mother, sister, brother)	Yes	62 (11.2)
	No	494 (88.8)
History of using assisted reproductive techniques in first-degree relatives	Yes	42 (7.6)
	No	514 (92.4)
History of oocyte donation	Yes	1 (0.2)
	No	555 (99.8)
History of blood donation	Yes	48 (8.6)
	No	508 (91.4)
Awareness of oocyte donation license (given by the Marja/Allamah)	Yes	81 (14.6)
	No	23 (4.1)
	I do not know	452 (81.3)
Awareness of oocyte donation	Very low	116 (20.9)
	Low	217 (39.0)
	Average	174 (31.3)
	High	49 (8.8)

The results showed that the mean ± SD scores of attitude to behavior, subjective norms, perceived behavioral control, and oocyte donation intention were obtained at 3.38 ± 0.42, 3.14 ± 0.54, 3.33 ± 0.69, and 2.64 ± 0.77, respectively. Spearman correlation test showed a positive correlation between awareness (*P* = 0.003, *r* = 0.127) and TPB constructs, including attitude toward behavior (*P* ≤ 0.001, *r* = 0.431), subjective norms (*P* ≤ 0.001, *r* = 0.413), and perceived behavioral control (*P* ≤ 0.001, *r* = 0.604), with the oocyte donation intention (Table 2).

**Table 2:** Mean, standard deviation (SD), and correlation between awareness and TPB constructs with oocyte donation intention

Variable	Mean ± SD	Spearman's correlation coefficient				
		**1**	**2**	**3**	**4**	**5**
1. Donation awareness	5.75 ± 2.72		0.202*	0.114**	0.253*	0.127***
2. Attitude to behavior	3.38 ± 0.42			0.407*	0.631*	0.431*
3. Subjective norms	3.14 ± 0.54				0.489*	0.413*
4. Perceived behavioral control	3.33 ± 0.69					0.604*
5. Oocyte donation intention	2.64 ± 0.77					

*P* ≤ 0.001^*^P = 0.007^**^ *P* = 0.003^**^

Regarding the predictive power of TPB constructs, the results of hierarchical linear regression model showed that among the components of the TPB, the most important predictors of oocyte donation intention in order of priority (based on standardized regression coefficients) included perceived power (Beta = 0.461), control beliefs (Beta = 0.154), and normative beliefs (Beta = 0.125). Accordingly, for each unit increase in the scores of perceived power, control beliefs, and normative beliefs (with the assumption that other components of the TPB model were constant), oocyte donation intention score increased by 0.503 (*P* < 0.001), 0.153 (*P* = 0.002), and 0.160 (*P* = 0.003) units, respectively. In total, 47% of the variance in the oocyte donation intention score could be explained by the components of the TPB model.

In the next stage, entering independent variables of spouse’s education, blood donation history, oocyte donation license (given by Marja/Allamah), and awareness (demographic variables that had *P* < 0.15 in univariate linear test) into the hierarchical multiple regression model, did not affect the significance of R2, the regression coefficients, and the significance of the model components ($\Delta$ R^2^ = 0.008, ∆F = 2.087, *P* = 0.081). In total, 47.4% of changes in the score of oocyte donation intention were explained by the model variables. Among the added demographic variables, only the spouse education variable predicted the oocyte donation intention (*P* = 0.015) and other variables had no effect. The most important predictors of oocyte donation intention (based on standardized regression coefficients) included perceived power (Beta = 0.459), control beliefs (Beta = 0.164), normative beliefs (Beta = 0.119), and spouse education (Beta = -0.076), by order of priority. Based on the results of the second stage, for each unit increase in the scores of perceived power, control beliefs, and normative beliefs (with the assumption that components of the TPB model were constant) oocyte donation intention score increased by 0.500 (*P* < 0.001), 0.163 (*P* = 0.001), and 0.152 units (*P* = 0.004), respectively. It was also found that the oocytes donation intention score was lower by 0.119 unit in individuals whose spouses had a university education, compared to those whose spouses had a non-university education (*P* = 0.015) (Table 3).

**Table 3:** Hierarchical regression analysis predicting oocyte donation intention by individual characteristics and TPB constructs

Step 1	**Predictors**		**B**	**Beta**	**S.E**	**t**	**P**
	Attitude towards behavior	Behavioral beliefs	0.017	0.009	0.078	0.220	0.826
		Evaluating behavioral consequences	0.024	0.024	0.055	0.415	0.656
	Subjective norms	Normative beliefs	0.160	0.125	0.053	3.007	**0.003**
		Motivation to comply	0.024	0.024	0.034	0.720	0.472
	Perceived behavioral control	Control beliefs	0.153	0.154	0.048	3.155	**0.002**
		Perceived power	0.503	0.461	0.050	9.995	** < 0.001**
Step 2	**Model Summary**: R^2^ = 0.476, $\Delta$R^2^ = 0.476, Adjusted R^2^ = 0.470, $\Delta$F = 83.564, *p* < 0.001						
	Behavioral beliefs		0.017	0.009	0.078	0.224	0.823
	Evaluating behavioral consequences		0.023	0.023	0.055	0.412	0.681
	Normative beliefs		0.152	0.119	0.053	2.859	**0.004**
	Motivation to comply		0.024	0.023	0.034	0.687	0.493
	Control beliefs		0.163	0.164	0.049	3.342	**0.001**
	Perceived power		0.500	0.459	0.050	9.968	** < 0.001**
	Educational level of husband: College ^a^		-0.119	-0.076	0.049	-2.441	**0.015**
	History of blood donation: Yes ^b^		0.107	0.039	0.086	1.251	0.212
	Issuance of permission to donate eggs by the imitation authority: Yes ^b^		-0.053	-0.024	0.073	-0.733	0.476
	Knowledge		-0.007	-0.024	0.010	-0.713	0.464
	**Model Summary**: R^2^ = 0.483, $\Delta$R^2^ = 0.008, Adjusted R^2^ = 0.474, $\Delta$F = 2.087, *p* = 0.081						

B, Unstandardized coefficients; Beta, Standardized coefficient; S.E., Standard Error

^a^reference category = High school or less

^b^reference category = No

## Discussion

The present study aimed to determine the predictors of oocyte donation intention using the TPB.

The results showed that the constructs of perceived power, control beliefs and normative beliefs were the strongest predictors of oocyte donation intention, respectively. Planned behavior theory constructs explained 47% of the variance of oocyte donation intention.

The results of the present study showed that most study participants had poor awareness of oocyte donation. A study conducted on the general population in Turkey (2006) showed that less than one-third of the participants had previous awareness about oocyte donation and its role in infertility treatment [21].

The knowledge about oocyte donation was demonstrated to be poor even among infertile populations under IVF treatment. In one study conducted on women undergoing assisted reproduction, 38% had “very little knowledge” of oocyte donation [22]. In another study (2017) performed on 69 women undergoing infertility treatment, 6% of patients “had never heard of oocyte donation” [23].

The results of this study showed a significant and positive correlation between the oocytes donation intention and the TPB constructs (including attitude toward behavior, subjective norms, and perceived behavioral control).

The results of a study that examined intentions and attitudes toward voluntary oocyte donation in the general population in the United Kingdom (2009) showed that oocyte donation intenders had a “more positive attitude toward oocyte donation” and perceived the “oocyte donation consequences” more favorably, compared to possible intenders. Possible intenders, in turn, had a more positive attitude, compared to non-intenders [11]. In a study conducted in Birjand, Iran (2016), a positive and direct correlation was found between attitudes, subjective norms, and perceived behavioral control to resolve the infertility problems, which was in line with the findings of the present study [18]. Consistent with the results of the present study, the results of a study performed in the United Kingdom (2006) showed that subjective norms and behavioral control affected oocyte donation intention [24]. Another study in Tehran, Iran (2014) showed that perceived behavioral control and subjective norms affected people’s willingness to donate blood, which was consistent with the obtained results in this study [25].

In the present study, Perceived behavioral control was perceived as the strongest predictor of oocyte donation intention. Perceived behavioral control describes the perceived ease or difficulty in practicing a behavior. In fact, if a person feels that s/he can overcome the external factors affecting oocyte donation, the oocyte donation intention will increase and they will deal more easily with the problems and complications that pursue. Therefore, perceived behavioral control in individuals needs to be increased to promote strategies for oocyte donation. In other words, to encourage people to donate oocytes; factors under their control should be considered. Based on the results of a study performed in Australia (2006) for the identification of factors explaining blood donation intention, the predictive power of perceived behavioral control and subjective norm were higher in the general population [26]. In studies performed in Birjand (2016) [18], and Mashhad, Iran (2013) [27], perceived behavioral control was the first and strongest construct related to the intention which was in line with the findings of the present study. Similarly, a study conducted in the United Kingdom (2006) showed that perceived behavioral control was the strongest predictor of oocyte donation [24].

In the present study, normative beliefs (a component of subjective norms) were the second predictor of oocyte donation intention. This finding was in line with a study in Ethiopia (2020) that stated that the subjective norm predicts the intention to donate blood [28]. Due to the specific culture, in some countries, including Iran, the person’s intention to behave (or one’s behavior) may be influenced by others’ expectations rather than what they really want to do. In this regard, the results should be interpreted considering the cultural or ethnic norms.

Therefore, to improve the practice of oocyte donation, interventions should target significant others (families, friends, relatives, and health professionals) as a whole, rather than focusing on individuals eligible for egg donation. If significant others are involved in oocyte donation, eligible individuals are more likely to engage in this behavior.

In the present study, the attitude construct was not a predictor of oocytes donation intention in women. Attitude is the degree to which a person has a favorable or unfavorable evaluation of a specific behavior.

The results of a study performed in Tehran, Iran (2019) indicated that the attitude was not a predictor of organ donation intention in participants [29]. Another study in the United Kingdom (2009) showed that attitude toward oocyte donation and oocyte donation outcomes were predictors of oocyte donation intention [11], which opposed the results of the present study.

The reason for the inconsistency between the results of different studies and the lack of predictive role of attitude in the current study may be due to the positive attitude of the subjects towards the egg donation method used in our study. Therefore, what can make a difference in women’s behavior and lead to oocyte donation behaviors is their control beliefs and perceived ability to perform the given behavior and overcome the potential obstacles, which are considered to be the constructs of perceived behavior control.

The present study showed that there was no difference in predicting the oocyte donation intention among people with different socio-demographic characteristics (except the spouse's education), as well as awareness of oocyte donation. These findings were in line with a study in Ethiopia (2020) that stated that prediction of intention to donate blood don’t vary among individuals having different sociodemographic characteristics and knowledge level towards blood donation [28].

Regarding the limitations of this study, one can refer to the online completion of questionnaires that can reduce the reliability of the data and makes it possible for any respondent to complete a questionnaire multiple times. Moreover, the fact that only people with smartphones and Internet access could complete the questionnaire reduced the participation chance for those lacking these facilities.

## Conclusion

The overall results of the present study showed that perceived behavioral control and normative beliefs (a component of subjective norms) were potential determinants of behavioral intention to donate oocytes in women of childbearing age. Implantation of intervention or training strategies in the field of oocyte donation requires the enhancement of social norms and perceived behavioral control in the population under study.

Propagation of normative beliefs in advertising media is a good strategy to promote oocyte donation as an accepted social value. Strategies should also be developed to remove barriers to action and create a higher understanding of control and focus on subjective norms.

## Data Availability

The dataset for this study is available from the corresponding author on reasonable request.

## Abbreviations

IVF: In Vitro Fertilization
TPB: Theory of Planned Behavior
CVR: Content Validity Ratio
CVI: Content Validity Index
VIF: Variance Inflammation Factor
